# Teaching statistics to medical students using problem-based learning: the Australian experience

**DOI:** 10.1186/1472-6920-4-31

**Published:** 2004-12-10

**Authors:** J Martin Bland

**Affiliations:** 1 Department of Health Sciences, University of York, Heslington, York YO10 5DD, United Kingdom

## Abstract

**Background:**

Problem-based learning (PBL) is gaining popularity as a teaching method in UK medical schools, but statistics and research methods are not being included in this teaching. There are great disadvantages in omitting statistics and research methods from the main teaching. PBL is well established in Australian medical schools. The Australian experience in teaching statistics and research methods in curricula based on problem-based learning may provide guidance for other countries, such as the UK, where this method is being introduced.

**Methods:**

All Australian medical schools using PBL were visited, with two exceptions. Teachers of statistics and medical education specialists were interviewed. For schools which were not visited, information was obtained by email.

**Results:**

No Australian medical school taught statistics and research methods in a totally integrated way, as part of general PBL teaching. In some schools, statistical material was integrated but taught separately, using different tutors. In one school, PBL was used only for 'public health' related subjects. In some, a parallel course using more traditional techniques was given alongside the PBL teaching of other material. This model was less successful than the others.

**Conclusions:**

There are several difficulties in implementing an integrated approach. However, not integrating is detrimental to statistics and research methods teaching, which is of particular concern in the age of evidence-based medicine. Some possible ways forward are suggested.

## Background

Problem-based learning (PBL) is a method of teaching and learning which is widely used in the education of medical students [1,2]. In problem-based learning, students working in a small group are presented with a problem, typically a description of a patient presentation. They decide what features of the problem are outside their present knowledge and divide these topics between them. They then research their topics using library and internet material and report back to the next small group tutorial with their findings. In a problem-based learning curriculum, this is the principal method of learning. More traditional methods, such as lectures and practical exercises, provide background and support material. An example of a PBL teaching problem is given in the Appendix.

This project was conceived at a meeting of statisticians from UK medical schools, which is held every year at the Burwalls conference centre, University of Bristol. In 2002, one of the topics for discussion was problem-based learning (PBL). Several things became apparent during this discussion. First, most of the statisticians present had no idea what problem-based learning was. Second, in the few UK medical schools where PBL was in use, statistics and research methods were not being taught through this medium. Statisticians in some of these schools felt excluded. Third, the new medical schools which were starting up in the UK were adopting PBL as their method of teaching.

As far as I know, only four of the older UK medical schools have adopted a PBL curriculum at the time of writing: Liverpool, Manchester, Glasgow, and St. George's. St. George's is a special case, as it has two medical courses. The five-year undergraduate entry course is taught in the traditional way, with a small 'case-based learning' element added on. The new four-year Graduate Entry Programme (GEP) is based on the McMaster model, a PBL course which takes graduates in any subject [2]. In none of these courses are statistics and research methods taught as an integral part of the PBL. At St. George's, for example, there is a parallel, non-PBL, seminar-based course. This attempts to match the illustrative material to the case of the week, but is taught separately from that case.

New medical schools, such as Anglia, Peninsular, and Hull-York, are being set up as PBL courses [3]. This is particularly suitable for courses based in more than one university, as in Peninsular (Plymouth and Exeter) and Hull-York. Problems can be set by teachers in either centre, and presented to students in both.

The adoption of PBL in new medical schools may indicate the possibility that PBL will become more widely adopted in UK medical schools. If this were to happen, there would be a real danger of statistics and research methods being marginalised in the medical curriculum. It is difficult enough to persuade medical students of the importance of these topics and it would be even more so if it were taught outside the mainstream of the course. More importantly, in the era of evidence-based medicine these topics should be central to the medical curriculum, not on the sidelines.

## Methods

During a sabbatical visit to Australia, I spent one to three weeks each at most of the medical schools. I interviewed educationalists and statisticians at each of these to ask how statistics and research methods were taught to medical students in their problem-based learning curricula. I had no prior knowledge of how this might be done, so the interviews were open in nature, rather than structured. I enquired about statistics as a subject in its own right and the wider principles of research, critical reading of research, and evidence-based medicine. I then synthesised the information collected to provide a picture of the current situation.

I visited the following universities with medical schools: the University of Western Australia, Perth, Flinders University, Adelaide, Monash University, Melbourne, the University of Melbourne, the Australian National University, Canberra (medical school about to commence), the University of Sydney, the University of Newcastle, and the University of Queensland, Brisbane. I omitted the University of Adelaide, the University of New South Wales, and James Cook University, Townsville, but was able to get information from them by email. I identified potential informants from the university website and emailed them as follows: 'I am interested in the teaching of statistics to medical students. Last year I visited several medical schools in Australia, but unfortunately I did not manage to visit [your university] on this trip, a serious omission. I have written a report on my experiences, which is available on one of my websites at . I am particularly interested in how statistics is taught in problem-based learning programmes. I am presenting this material at the forthcoming International Biometrics Conference in Cairns and preparing a paper for possible publication. I would be very interested to learn how these matters are ordered in [your university] . . . Are you the right person to ask? If not, could you suggest someone who would be?' I obtained helpful replies from all three universities.

## Results

I went to Australia in search of the fully integrated teaching of statistics and research methods as part of the PBL tutorials. I did not find it anywhere. I did find three different models:

• Material integrated but separately taught,

• A parallel course,

• PBL used only for 'public health' related subjects.

In addition, one school (New South Wales) did not use problem-based learning and one (James Cook) taught virtually no statistics. More information about the individual medical schools is available [see Additional file 1].

### Material integrated but separately taught

This approach was used or planned at the University of Sydney, the University of Melbourne, and the Australian National University. However, of these only the University of Sydney had actually put this into practice. The University of Melbourne and the Australian National University were about to implement what was essentially the Sydney model.

In this model, statistics and research methods are taught by PBL and the PBL triggers are integrated with the PBL problems for other parts of the course, but the material is not taught in the same tutorials or by the same tutors as anatomy, biochemistry and physiology. There will be a separate set of triggers for the statistics, etc., presented in a separate tutorial, and by separate tutors.

I asked why the main PBL tutors could not do this. As I understood it, the function of a PBL tutor is to facilitate and guide the group, not to impart knowledge. I had no problem, at least that I was aware of, in acting as PBL tutor when students were working with triggers designed to elicit questions about anatomy, biochemistry, and physiology, subjects of which I know virtually nothing. Besides, many of these tutors must routinely read journals which bristle with P values, t tests, correlation coefficients, etc. They must be familiar with the terms, if nothing else. Answers to this included:

• the tutors themselves refused to do it,

• in the early years of the course we need expert tutors,

• many tutors are still rooted in the old paradigm and are reluctant to embrace EBM and it was the view of my informants that experience round the world shows that it is difficult to teach EBM principles.

The team at the University of Sydney were on the whole positive about their course.

### A parallel course

This approach was used at Monash University, the University of Queensland, Flinders University, the University of Newcastle, and the University of Adelaide.

In this approach, a non-PBL course is given separately from the main PBL course. This may consist of any combination of lectures, seminars, practicals, web pages, or text handouts. Usually there is an attempt to link this to the PBL cases by using examples related to the case of the week. For example, the case of the week might be asthma and the parallel course could include a critique of a paper reporting a trial of a treatment for asthma.

There are several problems with this approach to teaching statistics and research methods. As noted in the Background, the subject may seem peripheral to the main thrust of the medicine course. Student feedback tends to give a much lower approval to parallel courses than to the main PBL teaching. Finally, teaching is dependent on the cases chosen by the PBL teachers, who may change the cases or reorder them at little or no notice. This can make statistics teaching, which is much more dependent on the order of presentation than most subjects in the medical curriculum, extremely difficult.

Most people involved in these courses were unhappy with them, the exception being a group project in the Flinders course.

### PBL used only for 'public health' related subjects

This approach was used at the University of Western Australia.

This was an unusual model, found at only one university. PBL teaching had been initiated by an enthusiast, after a period spent at McMaster University. She was a member of the public health group and persuaded her colleagues to introduce PBL. However, only about one third of the course is taught this way, anatomy, biochemistry and physiology are taught traditionally. The consequence of this approach is that the tutors are drawn from the population medicine area and so are quite happy to teach statistics, research methods, and EBM. The triggers can be chosen as population-oriented problems, rather than being restricted to the patient case.

People I spoke to were very positive about this course, not surprisingly as they saw themselves as the educational leaders in their institution.

## Discussion

I did not to find anywhere in Australia a single instance of truly integrated teaching of statistics and research methods through PBL. I have to conclude from this that there are considerable difficulties. I did, however, find an instance of unintegrated PBL teaching, at the University of Western Australia, where anatomy, biochemistry and physiology have continued to be taught in a more traditional manner.

What are these difficulties?

• If tutors are drawn mainly from the laboratory disciplines, they may be unsympathetic to the population and clinical foci of EBM and its core subjects. We need strong advocates to persuade them of the importance of these topics.

• If tutors are drawn from the clinical staff, they may be unsympathetic to the idea of EBM as a core activity. Converting the existing clinical teachers to the new paradigm of EBM may take a generation, but this will be a problem whatever teaching method we use.

• Tutors may be ignorant of the principles and details of statistics. This may be true. My own experience as a PBL tutor has been that lack of knowledge has seldom been a problem. It is not the function of a PBL tutor either to impart knowledge or to explain concepts. Persuading tutors of this should be part of their general PBL training.

• The changing patterns of cases as the course develops is a particular problem for statistics. This is undoubtedly true.

• The nature of the subject does not lend itself to PBL. It would certainly be difficult to deduce the calculations required for a t test from a problem based on a patient with asthma. However, we must consider what we actually want to teach. I do not think there is much point in teaching undergraduate medical students to analyse data, even using computers. What we need to teach them is how to understand research publications, the evidence on which we hope their future evidence-based practice will be based. We can certainly do this using triggers such as published papers, as the University of Western Australia has demonstrated.

• The patient case is not suited to teaching these subjects. This is true, though I do not think it is beyond us to devise cases which lead to research and evidence questions. However, we must persuade our colleagues that the patient vignette is not the only type of problem which we can use.

We should not see only the difficulties, however, but also the opportunities. If we can integrate statistics and research methods into PBL, there may be great advantages. One consequence of integration would be that the subject would not be marginalised or seen as separate. It would be just one aspect of medicine. Indeed, one of the features of a PBL course is that the distinctions between the different subjects should blur.

A second advantage would be that students would be learning statistics and research methods in the contexts in which we hope that they will apply them: the interpretation of clinical data and the assessment of research evidence. The relevance of the subject should be very clear and during their professional careers they would be more likely to be able to recall and use this material when needed.

So how can we do it? There must be an advocate for statistics and research methods at the start of the preparations for PBL, who is able to argue convincingly for the inclusion of these subjects. They must form part of the matrix of topics and problems. This is not an easy task and I have been told of great difficulty experienced at this level, as other teachers sought to exclude these subjects so that their own discipline could have more time. This is a natural human reaction, of course. I think that statistics and research methods are central to medical education, and would argue for more of them, at the expense of some anatomy if necessary.

We must get away from the idea that the problem must be a patient vignette. There are some statistical topics which can be covered quite conveniently in this way, such as measurement error, coefficients of variation, reference intervals, sensitivity and specificity, etc. After all, when my GP looks at my serum cholesterol on his practice computer, it has by the side of it a 95% reference interval. However, problems could equally be a published paper. We use these routinely in the teaching of statistics, research methods, and critical appraisal.

We could use a paper as a trigger for non-research methods topics. For example, a paper on an asthma trial could trigger questions about asthma as well as about randomisation. This may lead to too many questions being raised by the trigger. Another possibility would be to have such a trigger immediately following a case vignette problem on the disease in question. The patient vignette would raise the questions on anatomy, biochemistry, pharmacology, etc., associated with the disease. The following problem using a research paper would then raise only the research methodological questions. The fixed resource sessions in this week would then be devoted to these.

We may also, for variety, link research publications to the patient vignette by devices such as newspaper articles which the patient presents to the physician (e.g. reporting a trial), or say that in a patient problem the clinician has already found a Cochrane review.

Fixed resource sessions could include lectures, but I am very reluctant to suggest formal lectures in statistics and research methods for medical students. I would prefer to offer open question and answer sessions, where students can ask the statistician lecturer to explain anything they are unsure of. For example, if the PBL trigger is a randomised controlled clinical trial with results presented in terms of P values, we might be expecting this trigger to lead to questions such as 'why randomize?', 'what does P < 0.05 mean?', and 'should patients be told they are in a trial?'. Students should have made some attempt to answer these before the fixed resource session.

## Conclusions

There are several difficulties in implementing an integrated approach. However, not integrating is detrimental to statistics and research methods teaching, which is of particular concern in the age of evidence-based medicine. I remain optimistic that we can incorporate statistics and research methods into a fully problem-based curriculum. I think that if we do not, medicine will be the poorer for it.

## Competing interests

The author(s) declare that they have no competing interests.

## Authors' contributions

J M Bland is the sole author.

## Appendix

The following is a typical example drawn from the teaching at St. George's Hospital Medical School. The students work in a small group with a tutor to act as facilitator. The students are given the following information:

'You are a member of the on-duty Trauma Team in the Accident and Emergency department one Monday evening, when you are alerted to the imminent arrival of an "RTA (road traffic accident) patient". As she is being wheeled in to the Emergency Room, the paramedic accompanying her reports on the circumstances:

'The patient is called Janet Phillips, she appears to be a medical student in her early 20s, and was on a Pelican crossing when she was struck by a car which had gone out of control. Janet had been thrown some distance from the car by the force of the impact. She is confused, in shock, has superficial lacerations to her face and severe pain in her pelvis and legs.

'At the accident site her airway was cleared, oxygen administered, a traction splint applied to her right leg, and she was immobilised on a long spinal board.

'The driver had only minor injuries but his breath smelled strongly of alcohol, and he was now in police custody.

'In the Accident and Emergency Department Janet is given a pelvic clamp to arrest her internal bleeding.'

The students are told that they should address four general themes in their discussion: basic and clinical sciences, patient and doctor, community and public health, and professional development. In the first tutorial, they are asked to work through the following steps:

1. Clarify any terms and concepts in the scenario with which they are not familiar.

2. Define the problem(s) and issue(s) raised by the scenario.

3. Analyse the problems and issues, seeking explanations or hypotheses.

4. Agree on specific questions (learning objectives) for each of the four general themes to which you need answers.

They then work individually on tackling the questions agreed upon. In thesubsequent tutorial they discuss their findings and decide how the problems and issues raised by the scenario could be resolved. They may then be given further information about the patient, which might raise further topics for their research. When a problem has raised all the points required, a fresh problem is presented to the students.

## Pre-publication history

The pre-publication history for this paper can be accessed here:



## Supplementary Material

Additional File 1 'The Australian Universities visited'. This provides a descriprtion of teaching at the universities visited as part of this project. Click here for file
