# Supplementary material for: Teaching statistics to medical students using problem-based learning: the Australian experience
Source: BMC Med Educ. 2004 Dec 10;4:31. doi: 10.1186/1472-6920-4-31 (PMC539273; doi:10.1186/1472-6920-4-31)
Supplement: Additional File 1 — 'The Australian Universities visited'. This provides a descriprtion of teaching at the universities visited as part of this project. [file 1472-6920-4-31-S1.pdf]

Additional File: the Australian Universities visited

I did not manage to visit all universities with medical degrees, though I did visit most. These accounts are my personal distillation of what I learned at them. I recorded a greatly varying amount of information, but wherever I went my informants were generous with their time and very willing to share their experience. The variety is entirely due to my note-taking.

University of Western Australia, Perth

The University of Western Australia has a six-year medical course with an intake of 140 students. Most of these are aged 17-18, but there are some mature students.

The curriculum is divided into two three-year parts, roughly corresponding to pre-clinical and clinical.

In the first three years, PBL is used only for part of the curriculum, those that we might think of as the public health or social aspects. These are collectively the Foundation of Clinical Practice and make up about 1/3 of the course. Anatomy, biochemistry and physiology are taught in the traditional way by the science faculty.

Typical problems deal with rural health and deprivation, aboriginal health, etc. Typical problems which bring in statistical aspects are a report on leukaemia in clusters (rates, P values, confidence intervals) and high blood pressure (intra- and inter-personal variation).

In the second three years, evidence-based medicine (EBM) is taught from start of year 4. The idea is to start with the use of synthesized evidence than move downwards to the individual study. There is a one-day EBM seminar in the first four weeks, which deals with review and meta-analysis, the basic summary statistics used, odds ratio, number needed to treat, etc. Then there are clinical PBL tutorials with the expectation that students will look at synthesized evidence. There is a new, voluntary EBM clinic. Students are encouraged to do wider searches and to present these with a critique. There are back-up question and answer sessions, which are much appreciated by the students. In the fifth year there are two-hour EBM seminars about three-weekly, criticising randomised controlled trials, etc.

Further comments from UWA are that is very important to make sure that the learning method is up front for the students, that they are familiar and happy with it. This is just as important as tutor training. There is a need continually to reinforce PBL ideas. We also need to develop PBL stimuli as the course progresses, or students become rather bored with it. Patient presentations can be used to enrich PBL experiences, as can varying the type of student response requested. An example at UWA is a court-room scenario used in the geriatrics block.

Flinders University, Adelaide

Flinders has a graduate entry, 3 year course, with an intake of about 100 students, including a substantial number of fee-paying overseas students, mostly from North America.

Flinders University has a PBL programme. Flinders helped St. George's to set up the Graduate Entry Programme, and our course is largely based on theirs. There is very little statistics included in their problems. At the time the course was set up, there was no strong advocate in the school for statistics and research methods, though as the

students are graduates we might expect that some or even most of them have covered these subjects already.

There is what is described as a statistics elective, consisting of statistical questions attached to the problems on their website. It is unclear how many students attempt these.

However, there is an extensive small-group project, in which students practice statistical and research methods skills. The project is carried out in a community environment in rural areas by students working in groups of 3 to 5. A list of projects of interest to the communities which students visit is compiled, and students choose one. They design the instruments, which might be a random or convenience sample, with face to face or telephone interviews, often using a mixture of quantitative and qualitative methods. The locations are small country towns 300-400 Km away from Adelaide. (These distances are unimaginable in a UK context!) The studies are design in consultation with the communities, and often receive considerable publicity in local media such as radio. About 30 students visit a community at once and the programme runs three times a year. Students have to produce poster of their study which can go back to the local community, and publication is encouraged. There is AU$1,000 prize for the best in the year. Communities value the students contribution and when students talk to the stakeholders in the communities they see that their efforts are going to make a difference, which is very motivating. Studies can build upon previous projects and there is feedback.

Ethics approval can be a problem with medical students' projects. The Ethics Committee reviews proposals in a block after review by the tutor, who also ensures approval by stakeholders.

These projects are seen as fitting closely with the PBL philosophy, as students are presented with a problem which they have to solve. Some sample projects were:

- Health related volunteering in Port Pirie
- Are you healthy enough to do your work? (health worker survey)
- Health of workers in viticulture in Port Pirie
- How Port Pirie Regional Health Services are utilised and rated by residents
- Knowledge of Q fever in the Greater Green Triangle
- Riverland Mental Health Services: the youth perspective
- Aboriginal diabetes: closing the gap

Monash University, Melbourne

The course at Monash is in a state of transition from the previous non-PBL 6-year curriculum to a new PBL 5-year curriculum. The intake is about a total of about 140 Australian and 70 Malaysian students, mostly aged 17-18.

In the new curriculum, now in its second year, statistics is not taught as part of the PBL, but as a parallel course. Integration is greatly emphasised and so biostatistics and epidemiology are taught in an integrated way. There is a substantial practical and seminar based course in year one. There is only one lecture to orient to students in week 1. Epidemiological material was provided on a CD ROM, but this was reading rather than interactive. Statistical material was given on paper and much of the epidemiology was given on paper also, as it was needed in class. Students got a block

of material covering four weeks at once. The student response to the CD was discouraging, few seemed to use it.

The course is taught to 16 groups of about 14 or 15 students. This is done by having 8 groups on two different days, because of competition for tutors with other courses. Tutors are mostly epidemiologists, including Ph.D. students, about half are medically qualified but only one is statistically qualified. Most have a health sciences background, MPH, etc. It is a 2 hour tutorial, including a half hour break for coffee.

This is a summary of the course as it ran in the first year of the new curriculum:

Week 1. Opening lecture to entertain and motivate the students. Students were introduced to the idea of epidemiology using identification of HIV and AIDS (extract from the television film "And the Band Played On"), given a lecture on why statistics is important in medicine, and given a quiz on their current knowledge.

Teaching in the following weeks is seminar based, students told to do reading in advance, most don't.

Week 2. Exercise, mostly calculations based on a document on bites and stings. The document was long.
Week 3. Rural visit. (Students got back having forgotten the start of the statistics and epidemiology course.)
Week 4. Making measurements, external and internal rotation of hip. Distribution and measurement. Paper on prevalence of osteoarthritis, cross-sectional studies. This was a long paper, 14 pages of A4 print.
Week 5. Standard error, random sampling, using last week's data as a population. A practical exercise which didn't work very well. Scenario of occupational health rather than a full paper, concepts of "normal" and reference ranges.
Week 6. Confidence intervals and significance tests, t distribution, measures of association: relative risk and odds ratio, cohort studies. Paper reading exercise. Too much statistics for one session!
Week 7. Proportions and two-way tables, case-control studies, exercise on case-control paper.
Week 8. t tests, ethics of trials. Paper using t tests, critiques of randomised controlled trials using abstracts.
Week 9. Diagnostic tests, paired t tests, McNemar's test.
Week 10. Outbreak epidemiology.
Week 11. Correlation and regression, association and causation. Example on shingles.
Week 12. Non-parametric methods, exposure assessment.
Week 13. Revision.

Topics are all linked to the case of the week, though students seldom appeared to make the link.

Assessment is integrated. There are two mid-semester tests, MCQ and short answer, covering the whole course. At the end of the year there is an OSCE, totally integrated with a case-based structure.

The organiser, statistician Rory Wolfe, commented "If you lose the students early they will ignore the subject and concentrate on anatomy, etc. Integrated assessment allows this. There is too much work in the course to make it worth their while, if they take a strictly practical view. We are trying to teach material not core to students' interests."

Plans for a change of the running order of cases the second time the course is run are causing problems. This is currently under discussion. The order of presentation of topics in statistics is difficult to change and it seems likely that unless the team are going to rewrite most of the statistics course the notional link with the case of the week will be broken. They will be running with the same course in the second year.

In the first run, this course did not get as good feedback from students as other parts of the first year. David Goddard, a tutor on the course, told me that when they reached standard error and confidence intervals, his students rebelled. David just could not find the right words to help them. He then produced his own handout. Things improved and this handout was widely copied. He thought that this course was a moderate education experience, many students were not enthused.

What will happen in later years of the 5 year programme? There are four themes running through the curriculum, and statistics and research methods come under Population Health. In year 2 this includes knowledge management (where you get information from and whether you believe it, done by the Institute of Health Services Research at Monash), health economics, and health promotion. In 3rd year they will study critical appraisal, linked to the case of the week. In the 4th year the students will study preventive medicine and health service management.

David Goddard is a dedicated teacher with a lot of good ideas. He challenges the students by asking whether they want their careers to be as leaders or followers of opinion. David has many ideas for involving students in the classroom, such as a bingo game where small groups of students compete for a little prize. This uses numerical questions and gets collaboration going, bringing out the competitive spirit. Another is a crossword against the clock. He likes to challenge them with questions like "How would the world be worse off without P values?"! Well, how would it?

University of Melbourne

The University of Melbourne has a six-year programme with an intake of 270 students, 2/3 aged 17-18, 1/3 graduates. Some graduates can skip a year, the advanced medical science year in semesters 6 and 7, when students can choose from a great variety of subjects to study in depth, laboratory subjects, anthropology, medical humanities, etc.

A PBL course has been running for about five years. In the first 5 semesters, basic medical sciences are taught via PBL tutorials, using two tutorials per problem. At present public-health-related areas, including statistics, research methods, and evidence-based medicine, are not taught as part of the PBL programme, and consistently get lower student ratings than the PBL subjects. This course is currently undergoing considerable revision.

The new plan is to embed these topics in the PBLs, integrated with them but not taught by the same tutors. There will be a separate tutorial for each problem, where additional material related to the problem will be presented to the students. This second tutorial will be taught by "community oriented" tutors, from public health, general practice, etc. These sessions will be devoted to teasing out non-biological science issues. There will also be separate fixed resource sessions (e.g. lectures).

In semesters 8-12, much teaching is problem-based, but not PBL in the true sense, sessions are more tutor-oriented. Tutors in this clinical period have reported that students who have been through the PBL programme are much more independent in their thinking than students taught under the old curriculum.

Australian National University, Canberra

The Australian National University currently has no medical degree, but some students from the University of Sydney are taught at Canberra Hospital during their clinical period. However, the University is about to embark on a new medical degree, and the intake of Sydney students will be phased out as the course takes its first students in 2004.

The new course will be PBL, only the first two years have been planned. They will be using individual patient problems, with EBM in the clinical skills theme.

University of Sydney

The University of Sydney has a four-year course with an intake of 240 students, all graduates. The course is PBL, with three tutorials per problem. Problems are mostly individual cases, mostly set in a GPs surgery. The course is highly web-supported. All case material is presented on an intranet via terminals in the tutorial rooms. These include many still images with voice-over. A lot of resources are provided throughout the week. Apart from search facilities, there is a set of learning topics, a 1-2 page overview on the web as a kick start. There are up to 6 lectures and other theme sessions (labs, communication skills, etc.) but no dissection. The web-based nature of the course makes continual revision a relatively straightforward task.

EBM activities are included in the cases, specifically given this title. There are EBM add-ons to some of the cases and tutors are asked to point out to the students that there is an EBM activity for this case. Clicking the EBM box then brings up the related EBM activity.

In later years there is an "old-fashioned" clinical epidemiology course, using lectures and tutorials with EBM tutors. The sessions are linked to the case of the week.

Assessment at the end of the first year is formative only, at the end of the second year it is summative. The assessments include both written and practical tests, small clinical problem scenarios and multiple choice questions. A journal article is given to students two weeks ahead of the assessment.

One pre-registration task at the end of the course is to ask a patient "What do you really want to know?" and get an evidence-based answer, which is marked by the patient!

University of Newcastle

The University of Newcastle has a mixed intake of graduate and non-graduate students.

The University of Newcastle was the first medical school in Australia and one of the first in the world to adopt PBL. Currently, they have two PBL tutorials per week with a separate biweekly public health tutorial. In the first year the public health tutorials include elements of study design, in the second year they cover basic biostatistics and decision making, sensitivity and specificity, etc. The public health trigger material is matched to the case of the week.

University of Queensland, Brisbane

The University of Queensland in Brisbane has a graduate entry course, which uses problem-based learning. Evidence-based medicine is taught in a parallel stream to the PBL course, using material linked to PBL cases. There are 240 students per year, and

they hare taught in EBM tutorials by about 10 tutors. Tutors are from public health or EBM backgrounds. There are about 25 students per tutorial group, though not all attend.

It was assumed at the outset that being graduates the students would be familiar with statistical concepts, but this proved not to be the case. Thus teaching on P values, etc., had to be included in the tutorials.

The general view is that there is great rigidity in approach in the course. They tried to get EBM integrated into PBL in the initial planning, but failed. There were many objections to non-biological content and were unable to get material included. At one stage there was to be one lecture on EBM as the entire course! This led to the development of the present parallel course.

A typical example: in a case on deep vein thrombosis, the EBM content was to be the inclusion of a couple of abstracts on aspirin and on flight socks as prophylaxis during long-distance air journeys. This was vetoed on the grounds that providing these abstracts would not be PBL but didactic teaching! Of course, the abstracts are not the answer to the question on whether the a patient should take aspirin or wear flight socks, but the triggers for questions about the acceptability of the research evidence.
